# Auxin and Target of Rapamycin Spatiotemporally Regulate Root Organogenesis

**DOI:** 10.3390/ijms222111357

**Published:** 2021-10-21

**Authors:** Xiulan Xie, Ying Wang, Raju Datla, Maozhi Ren

**Affiliations:** 1 Labarotary of Space Biology, Institute of Urban Agriculture, Chinese Academy of Agricultural Sciences, Chengdu 610213, China; xiexiulan01@caas.cn (X.X.); wangying08@caas.cn (Y.W.); 2 Zhengzhou Research Base, State Key Laboratory of Cotton Biology, School of Agricultural Science of Zhengzhou University, Zhengzhou 450000, China; 3 Hainan Yazhou Bay Seed Laboratory, Sanya 572025, China; 4 Global Institute for Food Security in Saskatoon, University of Saskatchewan, Saskatoon, SK S7N 0W9, Canada

**Keywords:** auxin, TOR, crosstalk, embryonic root, primary root, lateral root, adventitious root, organogenesis

## Abstract

The programs associated with embryonic roots (ERs), primary roots (PRs), lateral roots (LRs), and adventitious roots (ARs) play crucial roles in the growth and development of roots in plants. The root functions are involved in diverse processes such as water and nutrient absorption and their utilization, the storage of photosynthetic products, and stress tolerance. Hormones and signaling pathways play regulatory roles during root development. Among these, auxin is the most important hormone regulating root development. The target of rapamycin (TOR) signaling pathway has also been shown to play a key role in root developmental programs. In this article, the milestones and influential progress of studying crosstalk between auxin and TOR during the development of ERs, PRs, LRs and ARs, as well as their functional implications in root morphogenesis, development, and architecture, are systematically summarized and discussed.

## 1. Introduction

All vascular plants have root systems including embryonic roots (ERs), primary roots (PRs), lateral roots (LRs), and adventitious roots (ARs) [1], all of which are required for fixing plants, absorbing water and nutrients, and storing photosynthetic products. The roots share similar structural features across plant species such as root caps, meristem zone, elongation zone, and maturation zone. The ERs develop from a zygote acting as totipotent stem cells. The PRs are derived from the ERs exhibiting geotropism and pluripotency. The LRs are derived from the pluripotent stem cells of the PR cambium, xylem parenchyma, and pericycle cells. ARs are differentiated from unipotent stem cells and existing stems, leaves, old roots, or hypocotyls, which are typically induced and activated by various stresses. They then undergo cell fate transitions from unipotent stem cells to pluripotent stem cells for de novo root organogenesis, which is widely used in the asexual reproduction of horticultural plants, forest trees, and medicinal plants. The accumulated data demonstrate that plant hormones are the most important regulators of root development, while auxin is the major player. Studies have demonstrated that target of rapamycin (TOR) signaling also plays key role in root organogenesis.

Auxin in an important plant hormone proposed to function as a morphogen with important regulatory functions during plant embryo development [2], organogenesis [3], gravitropism [4], apical dominance [5], the flowering [6], and stress response [7,8]. The regulatory effects of auxin are primarily orchestrated through local biosynthesis, polar transport, and signal transduction [9]. In the auxin local biosynthesis, the indole pyruvic acid (IPyA) pathway dominates in higher plants and is the most extensively investigated. In this pathway, tryptophan aminotransferase of Arabidopsis 1/tryptophan aminotransferase-related (TAA1/TAR) converts tryptophan to indole-3-pyruvic acid (IPyA), and then IPyA synthesizes indole-3-acetic acid (IAA) under the catalysis of flavin monooxygenase (YUCs) [10,11,12,13,14,15,16]. Auxin must be transported to specific parts to perform its corresponding functions. The gradient and asymmetric distribution of auxin in plants are crucial to the formation of plant root development patterns. This polar auxin transport is mediated by auxin influx and efflux facilitators, whose subcellular polar localizations guide the direction of auxin flow. Common auxin transport associated proteins primarily include influx carriers auxin resistant1/like AUX1 (AUX/LAXs), efflux facilitator PIN-formed (PIN), and ATP-binding cassette/multidrug resistance/P-glycoprotein (ABCB/MDR/PGP) with both influx and efflux functions [17]. In addition to local biosynthesis and transport, auxin signaling through receptors and downstream signal components is involved in the regulation of several developmental processes [18]. The transport inhibitor response 1/ auxin signaling F-boxes (TIR1/AFBs) protein is an important auxin receptor in *Arabidopsis*. TIR1/AFBs is an F-box protein and a component of SKP1, Cullin, and F-box complex (SCF), which is involved in protein degradation mediated by the proteasome. The auxin/indole-3-acetic acid (Aux/IAA) protein is a specific substrate of the SCF^TIR1/AFBs^ complex that negatively regulates auxin signal transduction. Aux/IAAs interact with auxin response factors (ARFs). ARFs are transcription factors involved in auxin-dependent transcriptional regulation. Post-translational modifications, particularly the reversible protein phosphorylation catalyzed by protein kinases and phosphatases, are also involved in the process of auxin biosynthesis, transport, and signal transduction [19]. In addition to natural auxin, some synthetic auxin analogs with similar structure and activities of endogenous auxin, such as 2,4-dichlorophenoxyacetic acid (2,4-D), naphthalcneacetic acid (NAA), 2,4,5-trichlorophenoxyacetic acid (2,4,5-T), dicamba, picloram, and quinclorac, have been developed and are used as growth regulators or herbicides in scientific research and commercial activities [20,21].

TOR is a phosphoinositide 3-kinase (PI3K)-related kinase [22] that is highly conserved in eukaryotes. It plays important functions in cell proliferation and growth by regulating protein translation, ribosome synthesis, and cell-cycle operation [23]. Clinically, mammalian TOR (mTOR) is an important target for disease treatment, and many familial cancers are related to misregulation of TOR [24]. In recent years, TOR has also attracted increasing attention from plant scientists. Of the components of the target of rapamycin complex 1 (TORC1), only the key genes including *TOR*, *lethal with SEC13 protein 8* (*LST8*), and *regulatory-associated protein of TOR* (*RAPTOR*) have been found in studied plants, while no members of the TORC2 complex have been observed in plants. The copy number of *TOR* differs in various species, but the structure of all TOR proteins is highly conserved. From the N-terminal to C-terminal, the TOR protein is organized into five domains: the HEAT repeat motif involved in protein—protein interaction, the FAT domain, the FRB domain for rapamycin–FK506-binding protein (FKBP12) interaction, the catalytically active kinase domain, and the FATC domain. The FRB and FATC domains play a key role in regulating kinase activity. During plant growth and development, factors such as nutrition [25,26], energy [27], hormones [28], and adversity [29] activate TOR kinase activity [30,31], while rapamycin, which is its inhibitor, is a secondary metabolite secreted by *Streptomyces hygroscopicus* in the soil. After binding to the FKBP12 protein, rapamycin–FKBP12 targets the FRB domain of the TOR protein to form a ternary complex that blocks TOR kinase activity and ultimately inhibits plant root growth [32]. In many plants, FKBP12 loses its binding function with rapamycin, resulting in a failure to inhibit TOR activity. Previous genetic results demonstrated that TOR promotes root growth [33]. For example, the homozygous loss-of-function *tor^−/−^* mutants in *Arabidopsis* display defects and lethality during early embryonic development [34]. Both RNA interference (RNAi)- and artificial microRNA (amiR)-mediated suppression of TOR activity showed growth defects in the roots and produced dwarf plants [35,36]. In addition, application of TOR inhibitors such as rapamycin, asTORis, and AZD8055 significantly reduced the growth of the PRs, LRs, and ARs [37,38,39], while the yeast and human FKBP12 overexpression transgenic lines were more sensitive to rapamycin showing similar growth phenotypes [33,40]. These findings suggest that TOR is involved in the regulation of root growth and development.

Several studies have used genetic and molecular approaches to identify functions of auxin [41,42] and TOR [43,44,45,46] signaling in the growth and development of plant roots. In recent years, various studies have further confirmed that TOR crosstalk with auxin mediates plant growth and development [47,48,49]. This review focuses on the interactions between auxin and TOR to regulate the development of plant ERs, PRs, LRs, and ARs.

## 2. Auxin and TOR Interplay Regulates the Formation of Embryonic Roots

**Figure 1:**
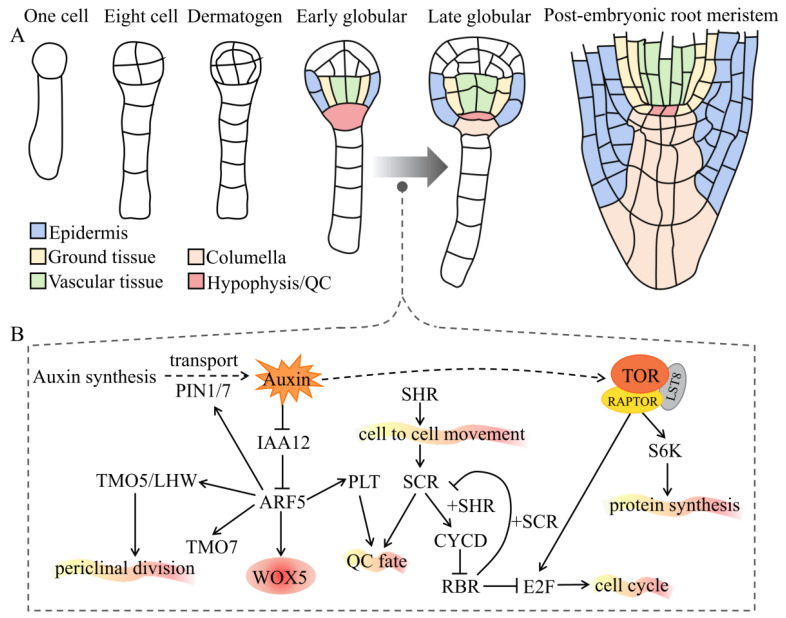
Auxin and TOR interplay regulates the formation of ERs. (**A**) The ontogeny of the root meristem during embryogenesis. Cell types and tissues are marked with different colors. Note that the hypophysis is specialized and divided into a quiescent center (QC) and columella in the early globular stage. (**B**) A schematic diagram for the molecular mechanism of how auxin and TOR work to regulate the formation of ERs. The straight arrows indicate direct positive regulation; the dotted arrows indicate indirect or multistep positive regulation; the T-shaped symbols indicate negative regulation. See main text for details. Abbreviations: ERs, embryonic roots; PIN, PIN-formed; IAA, auxin/indole-3-acetic acid; ARF, auxin response factor; TMO5/LHW, target of monopteros 5/Lonesome highway; WOX5, Wuschel-related homeobox 5; PLT, plethora; SCR, scarecrow; SHR, short root; CYCD, cyclin D; RBR, retinoblastoma related; E2F, early 2 factor; TOR, target of rapamycin; RAPTOR, regulatory-associated protein of TOR; LST8, lethal with SEC13 protein 8.

Root growth and development depend on the root apical meristem (RAM) established first during embryogenesis. In dicotyledonous plants such as *Arabidopsis*, the embryo development progresses through these key embryo stages: zygote, dermatogen, globular, heart, torpedo, bent, and mature [50,51,52,53] (Figure 1A). During the early embryonic development of plants, the zygote polarization, establishment of an apical–basal axis, dermatogen specialization, symmetrical mode transformation, hypophysis specialization, and root meristem formation all depend on auxin [54]. The formation of the root meristem, which is regulated by auxin and TOR (Figure 1B), is a key step for zygotic embryogenesis. Auxin synthesis in the integuments of the ovule is upregulated and transported to the apical domain of proembryo, leading to an increase in auxin levels and ultimately mediating early embryonic development [55]. During embryonic development, the root meristem initiates at the globular stage, where the auxin transporters PIN1/7 transport auxin to the suspensor cells, while the transported auxin accumulates in the uppermost cells of the suspensor [56]. However, auxin accumulation is insufficient to complete the establishment of hypophysis, and additional auxin-dependent signals are required. ARF5 (also called MONOPTEROS, MP) and IAA12 (also called BODENLOS, BDL) mediate downstream signaling as the core of auxin signaling pathway in *Arabidopsis* embryogenesis. The target of monopteros 5 (TMO5) and TMO7 are members of the bHLH transcription factor family and expressed in the hypophysis-adjacent embryo cells. These factors are necessary for IAA12/BDL-ARF5/MP-dependent embryonic root formation [57]. The TMO5/LHW dimers regulate periclinal division, vascular initial cell production, vascular cell proliferation, and xylem fate determination in the embryo and RAM [58]. More importantly, TMO7 is synthesized and localized to the hypophysis and plays a role in auxin-dependent ER formation [57,59]. In addition, ARF5/MP promotes the expression of PINs in the pro-vascular cells of globular embryos, resulting in auxin accumulation in the basal pole of proembryos [60]. The hypophysis cells divide unevenly, while smaller cells develop into a quiescent center (QC), which can maintain the stem-cell characteristics of the cells around the root meristem and subsequently differentiate into different tissues. *Wuschel-related homeobox 5* (*WOX5*) is a HOMEOBOX family gene specifically expressed in QC and plays a vital role in determining the fate of stem cells regulated by ARF5 [61,62,63]. Many transcription factors and proteins are involved in the development of the ERs in the later stage of embryonic development, including PLETHORA (PLT), SCARECROW (SCR), SHORT ROOT (SHR), and RETINOBLASTOMA RELATED (RBR). ARF5 activates the expression of the downstream target *PLT* gene [60]. *PLT* belongs to the AP2/ERF transcription factor family and plays a key role in the maintenance and specialization of root stem cells during embryonic development [64]. SHR is a key transcription factor for cell fate determination containing the GRAS domain and is expressed from the columella. Its protein migrates to the endothelium, where it interacts with another transcription factor, SCR, from the same family. It is mobilized into the nucleus by SCR, activates the expression of CYCD6;1, promotes the asymmetric division of the initial cells, and produces endothelial and cortical cell lines. CYCD6;1 phosphorylates RBR. The interaction of RBR and SCR inhibits the transcriptional activation of CYCD6;1 by SHR-SCR, thereby preventing further asymmetric division [59]. Generally, auxin local biosynthesis, polar transport (PINs and LAXs), reporter (TIR1/AFBs), and the downstream output of signal transduction (TMO5/7, SHR, SCR, PLT, and WOX5/7) work together to trigger the formation of ERs [65].

Due to the lethality of *tor^−/−^*, the studies of TOR-mediated embryonic development are much more difficult than those of auxin signaling during embryonic root formation. Several mutations interfere with normal auxin activity (such as *arf5*, *pin1*, *3*, *4*, *7*, *yuc1*, *4*, *10*, *11*) and have been shown to affect embryonic root meristem formation [66]. Furthermore, Menand et al. were the first to use forward genetics to isolate two T-DNA insertion mutants *tor1* and *tor2* from *Arabidopsis* in 2002; this homozygous mutant can lead to developmental arrest at the early embryonic stage [30]. Similarly, the mutation of *Atraptor1* the key subunit of TOR complex 1 and caused defects during early embryonic development in plants. It is phenotypically similar to *tor* [67,68]. TOR also directly regulates ribosomal protein S6 kinase (S6K) activity through phosphorylation, thereby regulating the protein translation or re-initiation of translation. Consistently, *s6k1*/*s6k2* has an embryonic lethal phenotype similar to *tor* [69]. Another study used chemical genetics to determine that *S6K2* overexpression can partially rescue the growth arrest phenotype caused by *tor* [39]. These observations imply that the TOR–S6K pathway mediates TOR regulation of the synthesis of related proteins during plant embryo development, many of which are related to auxin signaling. Overall, auxin and TOR signals share overlaps during the early embryonic development of plants and are vital to the formation of ERs.

## 3. Auxin and TOR Interact to Regulate the Development of Primary Roots

**Figure 2:**
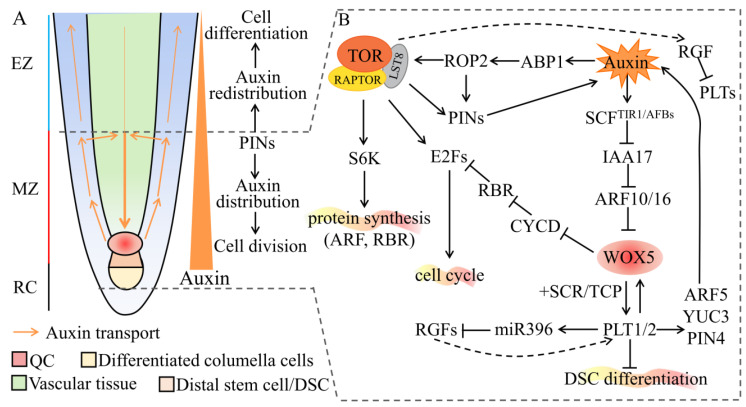
A schematic diagram of how auxin and TOR synergistically regulate the development of PRs. (**A**) The transport and concentration gradients of auxin in the primary RAM. Cell types and tissues are marked with different colors. Abbreviations: RAM, root apical meristem; EZ, elongation zone; MZ, meristem zone; RC, root cap; QC, quiescent center. (**B**) The molecular mechanism of auxin and TOR cross-regulate primary root development. The straight arrows indicate direct positive regulation; the dotted arrows indicate indirect regulation; the T-shape symbols indicate negative regulation. See main text for details. Abbreviations: PIN, PIN-formed; SCF, SKP1, Cullin and F-box complex; TIR1/AFBs, transport inhibitor response 1/auxin signaling F boxes; IAA, auxin/indole-3-acetic acid; ARF, auxin response factor; WOX5, Wuschel-related homeobox 5; PLT, plethora; SCR, scarecrow; TCP, teosinte-branched cycloidea PCNA; RGFs, root meristem growth factors; DSC, distal stem cell; YUC3, yucca3; ROP2, GTPase Rho-related protein 2; ABP1, auxin-binding protein 1; S6K, S6 kinase; CYCD, cyclin D; RBR, retinoblastoma related; E2F, early 2 factor; TOR, target of rapamycin; RAPTOR, regulatory-associated protein of TOR; LST8, lethal with SEC13 protein 8.

During post-embryonic seed development, the RAM is one of the main repositories of self-renewing stem cells, which provides new cells to support root growth. The longitudinal structure of the root is divided into the root cap (RC), the meristem zone (MZ), the elongation zone (EZ), and the maturation zone (Figure 2A). QC is located on the tip of the RAM, where stem cells are produced. The stem cells produce their progeny cells, which divide and differentiate, establishing the elongation zone [70]. Studies have demonstrated that the maximum concentration of auxin is closely related to root morphogenesis, while the establishment of an auxin concentration gradient can be achieved by the polar transport of auxin transport carrier PINs. The polar localization of PIN auxin efflux carriers is closely related to root gravitropism [71,72,73,74,75,76]. The auxin signal transduction elements IAA17 (also known as AUXIN RESISTANT3, AXR3) and ARF10/ARF16 negatively regulate *WOX5* transcription and restrict its transcript into QC, thereby inhibiting *PLT1/2* gene expression and mediating the differentiation of distal stem cells (DSCs). At the same time, PLT and SCR interact with plant-specific teosinte-branched cycloidea PCNA (TCP) transcription factors to directly regulate the promoter activity of the *WOX5* and maintain *WOX5* expression level in QC [77]. However, in transit-amplifying cells, *PLT* expression is restricted by root meristem growth factors (RGFs), while, in the stem-cell niche (SCN), with the highest *PLT* levels, *PLT* activates miR396 to degrade proliferation induction of the mRNA of *RGFs* [78]. This feedback mechanism helps maintain *PLT* expression levels around the SCN, forming a *PLT* concentration gradient consistent with the auxin concentration gradient. Furthermore, an RGF peptide gradient recognized by RGF receptors helps stabilize the *PLT* transcription factor gradient [79]. Interestingly, Santuari et al. found that auxin can induce *PLT* expression, while PLT also controls the synthesis, transport, and signal transduction of auxin. For example, PLT2 directly activates the gene expression of *PIN4*, *YUC3*, and *ARF5* [80]. In summary, the IAA–ARF, PLT–SCR–WOX5, and RGF–PLT signaling pathways are involved in auxin-mediated growth and development in plant PRs (Figure 2B).

Auxin can also interact with TOR to regulate the development of PRs through the ABP1–ROP2–PINs signaling pathway (Figure 2B). Auxin-binding protein 1 (ABP1) is one of the earliest identified cell surface receptors for auxin, which can bind to auxin and synergistically activate GTPase Rho-related protein 2 (ROP2). Activated ROP2 interacts with PINs on the cell surface to participate in endocytosis [81]. As described in the preceding part of the text, the PINs act as efflux facilitators, which are involved in the polar transport of auxin. Additionally, the auxin signal enhances ROP2 activity, which can bind to TOR and promote its phosphorylation [82]. TOR is a highly conserved master regulator of plant root development, which is involved in protein translation and the cell cycle. TOR can activate S6Ks via phosphorylation to control the protein translation process. Schepetilnikov et al. found that auxin activates the TOR–S6K1 signaling pathway, which leads to Eif3h phosphorylation. Phosphorylated Eif3h regulates mRNA containing upstream open reading frames (uORFs) to complete the translation re-initiation [83]. Another study demonstrated that the mRNA regulated by TOR–S6Ks includes auxin signaling pathway elements such as ARFs and RBR [69]. Therefore, auxin activates TOR–S6Ks, a signaling pathway that regulates auxin signaling protein synthesis. Early 2 factors (E2Fs) are key transcription factors regulating the cell cycle and are a direct substrate of TOR in plants. TOR can activate gene expression in the G_1_–S and G_2_–M phases by phosphorylating E2Fa and E2Fb, respectively [84]. In contrast, auxin-responsive WOX5 inhibits the QC cell division promoted by CYCD3;3 and CYCD1;1, and regulates the expression of CYCD3;3 in QC by directly interacting with CYCD3;3 promoters. Therefore, WOX5 can initiate and maintain the quiescent state of QC by excluding CYCD activity from QC and inhibiting cell division [85]. Additionally, through a large-scale screening of *Arabidopsis* mutants, Yuan et al. identified the auxin efflux protein PIN2 as a key downstream target of TOR kinase and found that glucose-activated TOR phosphorylates and stabilizes the PIN2 protein. This affects the gradient distribution of PIN2 in the PRs of *Arabidopsis* [86]. In summary, auxin regulates the activity of TOR kinase, while TOR determines the auxin maximum and its concentration gradient. They coordinate to regulate the development of PRs in protein synthesis, the cell cycle, and auxin transport.

## 4. Auxin and TOR Spatiotemporally Regulate Lateral Roots Organogenesis

**Figure 3:**
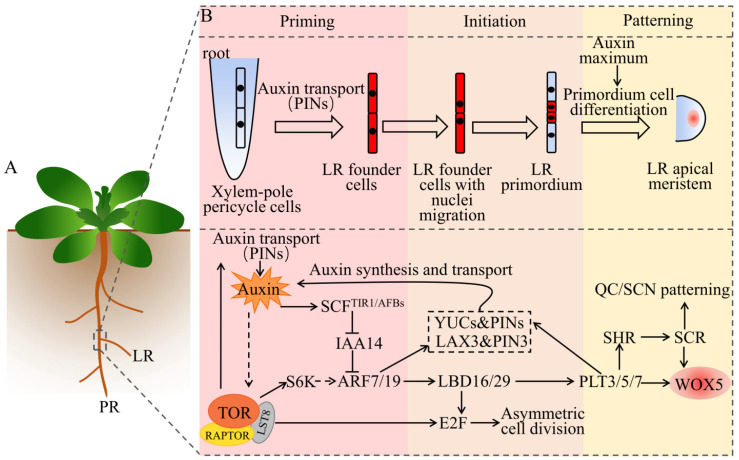
A schematic of the synergistic regulation of LRs organogenesis by auxin and TOR. (**A**) Models of different types of roots, using *Arabidopsis* as an example. PR represents the primary root, which develops from the ER. LR represents the lateral root, which develops from the PR. (**B**) The process and molecular mechanism of auxin and TOR synergistically regulate the development of LRs. In certain xylem-pole pericycle cells, the transport and perception of auxin specify a set of LR founder cells. The accumulation of auxin triggers the LR founder cells with nuclei migration, asymmetric cell division, and formation of LRP. The auxin maximum induces the division and differentiation of the LRP and forms an LR apical meristem. The straight arrows indicate direct positive regulation; the dotted arrows indicate indirect or multistep regulation; the T-shape symbols indicate negative regulation. See main text for details. Abbreviations: LRP, lateral root primordium; PIN, PIN-formed; SCF, SKP1, Cullin and F-box complex; TIR1/AFBs, transport inhibitor response 1/auxin signaling F boxes; IAA, auxin/indole-3-acetic acid; ARF, auxin response factor; LBD, lateral organ boundaries domain; YUC, yucca; LAX, like AUX1; WOX5, Wuschel-related homeobox 5; PLT, plethora; SCR, scarecrow; SHR, short root; QC, quiescent center; SCN, stem=cell niche; S6K, S6 kinase; E2F, early 2 factor; TOR, target of rapamycin; RAPTOR, regulatory-associated protein of TOR; LST8, lethal with SEC13 protein 8.

Evolved root branches, which are primarily LRs, can enhance the plasticity of plants [87]. LRs are mainly derived from pericycle cells, which are located in the outermost layer of the central column [88]. The formation of pericycle cells can maintain the ability of a cell to divide for a long time, allowing the plant to flexibly form LRs in response to environmental changes [89]. The formation of LRs includes several key processes (Figure 3): (i) priming of LRs: the Auxin is polarly transported to and accumulates in xylem pole pericycle cells, specifying a group of LR founder cells; (ii) initiation of LRs: the activated specified LR founder cells undergo nuclear migration, and then produce LR primordium (LRP) via asymmetric cell division [90]; (iii) patterning of LRs: in the LRP, the polar transport of auxin-mediated PINs forms an auxin maximum, which induces the division and differentiation of the LRP. In this way, the LRP passes through the endodermis, cortex, and epidermis of the primary root, until it breaks through the surface of the primary root to form LRs. Interestingly, the auxin maximum formed in pericycle cells plays a key role in the initiation of the LRP; however, auxin can only induce the formation of the LRP in the xylem pole pericycle. This indicates that auxin signaling and xylem pole pericycle cell specification both contribute to the initiation of the LRP [91].

Studies on *Arabidopsis* and other plant species have revealed the role of auxin in the formation of the LRs [92]. First, auxin signals are transported to xylem pole pericycle cells by AUX1 and PINs, which are sensed by the auxin receptors TIR1 and AFBs (AFB1, AFB2, and AFB3). Second, this auxin leads to the degradation of IAA14 (Aux/IAA repressor protein, also called solitary root, SLR) through the SCF^TIR1/AFBs^ complex and 26S proteasome. The de-inhibition of ARF protein activity (ARF7/ARF19) activates the target gene lateral organ boundaries domain 16/19 (LBD16/LBD29) and other target genes required for asymmetric cell division, initiating the formation of the LRP [93]. LBD16 (also called ASYMMETRIC LEAVES 2-LIKE 18, ASL18) is essential for LR initiation in *Arabidopsis*, which is a member of the LBD/ASL gene family encoding plant-specific transcription factors. Recent studies in legumes have found that LBD16 is involved in the formation of LRs and nodules, and the gene plays an important role in both processes [94,95]. Both ARF7 and ARF9 are required for LR formation, and the *arf7arf9* double mutant exhibits defects in LR generation [96]. The overexpression of *PLT3*, *PLT5*, and *PLT7* is sufficient to restore the blocking of the formation of LRP in the *arf7arf9* mutant, while, in the *plt3plt5plt7* triple mutant, the morphology of the LRP, the auxin response gradient, and the expression of the RAM marker genes are all affected [97]. These three *PLT* genes are downstream of the ARF7- and ARF9-mediated auxin-response pathway. As the downstream signal of ARF7/19, the auxin influx and efflux carrier proteins LAX3 and PIN3 are considered a feedback mechanism for auxin signal amplification [98]. Similar situations also occur as PLTs affect auxin synthesis genes YUCs and transport protein PINs [98]. Additionally, the findings of Berckmans et al. (2011) demonstrated that auxin-induced LBD18 and LBD33 could form a heterodimer and activate the expression of the transcription factor *E2Fa* [99]. Importantly, the loss-of-function mutant of E2Fa, E2Fa^ΔRB^, severely affects the development of the LRs [100]. Therefore, the ARF–LBD–E2F pathway uncovers a molecular mechanism that converts auxin signals into cell-cycle signals.

In legumes, the results of RNAi of TOR demonstrated that TOR inhibited LR elongation, changed the density, size, and number of root hairs, and reduced the expression of cell cycle-related genes *CyclinD1* and *CyclinD3* [101]. In *Arabidopsis*, the TOR inhibitor AZD8055 (1 μM) inhibits cell division in LR primordia and interferes with the stimulation of *Azospirillum brasilense* Sp245 (a symbiotic bacterium) on plants. Additionally, the *Azospirillum brasilense* mutant FAJ009 with impaired auxin synthesis fails to elicit TOR signals, displaying a phenotype similar to that of the wild type [102]. Similarly, studies in *Phaseolus vulgaris* also indicate that TOR is a key regulator of LR formation during mycorrhizal symbiosis [103]. In summary, the plant hormone auxin is essential for stimulating TOR signaling, while TOR also plays a pivotal role in the development of LRs in plants.

## 5. Auxin and TOR Synergistically Regulate the Regeneration of Adventitious Roots

**Figure 4:**
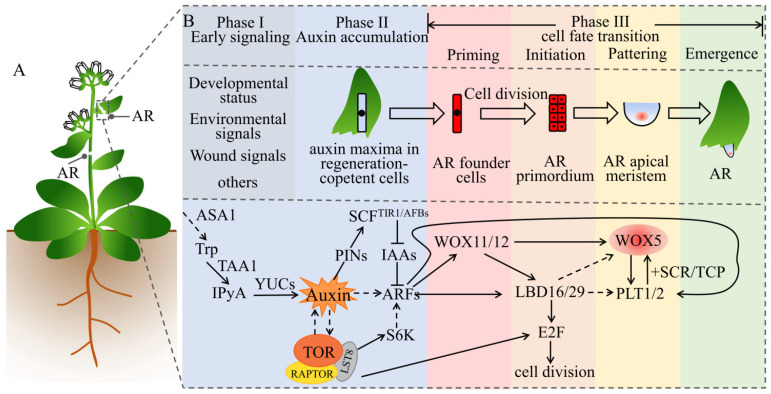
Auxin and TOR synergistically regulate the regeneration of ARs. (**A**) AR represents adventitious roots, which can be formed after leaves, stems, or hypocotyls are injured or can be formed on hypocotyls or hypocotyl–root nodes in response to environmental and/or developmental signals. (**B**) The process and molecular mechanism of auxin and TOR synergistically regulate the development of ARs. According toXu (2018) [105] and Yu (2017) [106], the organogenesis of ARs contains three continuous phases: early signaling, auxin accumulation, and cell fate transition. The cell fate transition is further divided into four stages: priming, initiation, patterning, and emergence. The straight arrows indicate direct positive regulation; the dotted arrows indicate indirect, multistep, or no direct evidence of regulation; the T-shape symbols indicate negative regulation. See main text for details. Abbreviations: ASA1, anthranilate synthase 1; TAA1, tryptophan aminotransferase of Arabidopsis 1; IPyA, indole-3-pyruvic acid; YUCs, yuccas; PIN, PIN-formed; IAA, auxin/indole-3-acetic acid; ARF, auxin response factor; S6K, S6 kinase; LBD, lateral organ boundaries domain; LAX, like AUX1; WOX, Wuschel-related homeobox; PLT, plethora; SCR, scarecrow; TCP, teosinte-branched cycloidea PCNA; E2F, early 2 factor; TOR, target of rapamycin; RAPTOR, regulatory-associated protein of TOR; LST8, lethal with SEC13 protein 8.

The ARs and LRs share key elements of genetic and hormone regulatory networks; however, their regulation mechanisms slightly differ. The regeneration of ARs is essential for survival after a serious wound and is considered to be a strong natural selective trait [104]. Plants can rapidly induce auxin biosynthesis at the stimulation site via signaling such as developmental status, environment, and wounding signals, which then trigger a signal cascade reaction to promote cell fate transition and produce ARs (Figure 4A). Xu et al. divided the de novo regeneration of ARs into three continuous phases: early signaling, auxin accumulation, and cell fate transition. Cell fate transition is further divided into four steps: priming, initiation, patterning, and emergence. This completes the transition from cells with regenerative capacity to fully form ARs [105,106] (Figure 4B). The entire process involves two types of cells with different functions: one is the converter cell that converts early signals (input) into auxin flux (output); the other is the regenerative competent cell that undergoes a fate transition under the guidance of auxin [105]. The protocambium cells and xylem parenchyma cells around the vascular tube at the stimulation site (stems and leaves) can regenerate. In vitro tissues stimulate the synthesis, transport, and accumulation of auxin in the body under stimulus signal induction. The auxin maxima activate the expression of the transcription factor *WOX11* and its homologous gene *WOX12*. This functionally upregulates LBD16 and LBD29, leading to cell fate transition in the parenchyma cells located in or near the phylloblastoma to the initiating cells of root regeneration. This clarifies the first step of the de novo regeneration of ARs [107]. WOX11/12 further activates downstream WOX5/7 [108] and LBD16/29 [107], while LBDs then activate the cell-cycle regulatory protein E2F [84]. In the *lbd16* mutant, the upregulation of *WOX5* and *PLT1/2* was slower, compared with the wild type [109]. However, there is currently no evidence that *PLT1/2* and *WOX5* are directly or indirectly regulated by LBD16. As such, LR founder cells are transformed into root primordium cells via cell division. This is the second occurrence of cell fate transition, after which the root primordium cells continue to divide and differentiate to form complete ARs. Therefore, auxin is responsible for the formation of ARs.

ARs exist in many plant and tree species in nature, such as poplar, willow, rose, holly, and chuanxiong, and they play an important role in asexual reproduction. Analysis of AUX/IAA gene family mutants has demonstrated that the mutants have no or reduced ARs similar to *iaa28*, *iaa14*/*slr1*, *iaa19*/*msg2*, and *iaa3*/*hy*. In contrast, overexpression of TIR1 and AUX1 promoted the development of ARs [110]. In poplar trees, the *Arabidopsis TIR1* homologous gene *PagFBL1* has been identified, and it is involved in the formation of ARs. Overexpression of *PagFBL1* stimulates adventitious root formation and increases root biomass, while knockdown of *PagFBL1* delays adventitious root formation and reduces root biomass. Further research found that *PagIAA28* is a downstream target of *PagFBL1*, while the PagFBL1–PagIAA28 module promotes the formation of ARs and can increase the reproductive efficiency of poplars via cuttings [111]. In the banyan tree, a comparative analysis of multi-omics data performed on aerial root types (*Ficus microcarpa*) and nonaerial root types (*Ficus hispida*) demonstrated that *PIN1*, *TAR*, *YUC*, *IAA14*, *ARF7/19*, *PLT2*, and *WOX11* were highly expressed in the RAM of the aerial roots [112]. Studies in potatoes found that TOR and auxin are required in adventitious root formation. The auxin receptor mutant *tir1* is sensitive to TOR inhibitors, while the double and quadruple mutants *tir1afb2*, *tir1afb3*, and *tir1afb1afb2afb3* are more sensitive to the rapamycin inhibitor asTORis than the single mutant *tir1*. Consistently, overexpression of AtTIR1 in *Arabidopsis* and potatoes can partially overcome the inhibitory effect of asTORis and promote adventitious root formation under asTORis treatment. These observations indicate that TOR signaling regulates adventitious root formation by mediating auxin signals in *Arabidopsis* and potatoes [38]. In conclusion, auxin and TOR are involved in the regulation of ARs regeneration in plants. Considering the conservative regulation of the TOR signaling pathway on protein translation and the cell cycle, the auxin and TOR synergistic regulation of ARs regeneration needs further study.

## 6. Outlook

Present studies have demonstrated that different types of plant roots share key elements of the genetic and auxin–TOR regulatory networks, including TIR1/AFBs–Aux/IAAs–ARFs, auxin–ABP1–ROP2, SHR–SCR–PLT, WOX5/7, PINs, CYC–RBR–E2F, and TOR–S6K/E2F/PINs. However, their specific underlying mechanisms differ slightly. Future studies related to TOR regulation induced by plant hormones will, therefore, be of great importance.

Additionally, studies have demonstrated certain basic principles, including the specialization mechanism of stem cells, cell fate determination, and cell reprogramming of plant roots. Studying plant roots can promote their application in agriculture and biotechnology. The “single tree makes a forest” of banyan tree, the “one bamboo makes a sea” of bamboo, and the deep rooting of alhagi (*Alhagi sparsifolia* Shap.) are examples of how powerful plant roots can be. The aerial roots of the banyan tree can grow on high-altitude branches far from the ground. They rapidly grow toward the ground until they penetrate the soil, where they quickly develop into supporting roots. This can reduce the risk of tree collapse and contribute to roots constantly expanding around the tree, making the growth of a forest possible. This mechanism has significant potential in urban agriculture, tropical agriculture, and leisure tourism agriculture. In contrast to the banyan tree, the ARs of bamboo grow underground; horizontal stems take root at the stem nodes. Eventually, the roots grow downward and connect with other roots through the underground creeping rhizome. The strong reproductive and regenerative ability of bamboo roots can form a dense forest of bamboo trees. The alhagi plant is a desert pioneer with a huge root system. Its primary roots can penetrate 20–30 m underground to reach water in arid environmental conditions. As such, alhagi is expected to become a pioneer plant on Mars and other desert planets, which could give it a central role in future Martian expeditions. There are countless plants with well-developed root systems in nature. An in-depth study of the key functional genes for the formation of these plant roots, a deep understanding of the biological mechanism of root development, and the use of advanced technologies such as synthetic biology and gene editing will all contribute to a better understanding of other economically valuable crops, such as fruit trees and other trees with significant agronomic value.

## Data Availability

Not applicable.

## Abbreviations

ABCB/MDR/PGP	ATP-binding cassette/multidrug resistance/P-glycoprotein
ABP1	auxin-binding protein 1
amiR	artificial microRNA
AR	adventitious root
ARF	auxin response factor
ASA1	anthranilate synthase 1
AUX/IAA	auxin/indole-3-acetic acid
AUX1/LAX	auxin resistant1/like AUX1
CYC	cyclin
DSC	distal stem cell
E2F	early 2 factor
ER	embryonic root
EZ	elongation zone
FKBP12	FK506-binding protein 12
IAA	indole-3-acetic acid
IPyA	indole-3-pyruvic acid
LBD	lateral organ boundaries domain
LR	lateral root
LRP	lateral root primordium
LST	lethal with SEC13 protein 8
MZ	meristem zone
NAA	naphthaleneacetic acid
PIN	PIN-formed
PLT	plethora
PR	primary root
QC	quiescent center
RAM	root apical meristem
RAPTOR	regulatory-associated protein of TOR
RBR	retinoblastoma related
RC	root cap
RGF	root meristem growth factor
RNAi	RNA interference
ROP2	GTPase Rho-related protein 2
S6K	S6 kinase
SCF	SKP1, Cullin, and F-box complex
SCN	stem-cell niche
SCR	scarecrow
SHR	short root
TAA1/TAR	tryptophan aminotransferase of Arabidopsis 1/Tryptophan aminotransferase-related
TCP	teosinte-branched cycloidea PCNA
TIR1/AFB	transport inhibitor response 1/auxin signaling F box
TMO5/LHW	target of monopteros 5/Lonesome highway
TOR	target of rapamycin
TORC	target of rapamycin complex
WOX5	Wuschel-related homeobox5
YUC	yucca

## References

[1] Ge Y., Fang X., Liu W., Sheng L., Xu L. (2019). Adventitious lateral rooting: The plasticity of root system architecture. Physiol. Plant.

[2] Winnicki K. (2020). The winner takes it all: Auxin-the main player during plant embryogenesis. Cells.

[3] Zhou P., Fatima M., Ma X., Liu J., Ming R. (2019). Auxin regulation involved in gynoecium morphogenesis of papaya flowers. Hortic. Res..

[4] Zhang Y., Friml J. (2020). Auxin guides roots to avoid obstacles during gravitropic growth. New Phytol..

[5] Balla J., Medvedova Z., Kalousek P., Matijescukova N., Friml J., Reinohl V., Prochazka S. (2016). Auxin flow-mediated competition between axillary buds to restore apical dominance. Sci. Rep..

[6] Zhang Y., Rodriguez L., Li L., Zhang X., Friml J. (2020). Functional innovations of PIN auxin transporters mark crucial evolutionary transitions during rise of flowering plants. Sci. Adv..

[7] Singh H., Bhat J.A., Singh V.P., Corpas F.J., Yadav S.R. (2021). Auxin metabolic network regulates the plant response to metalloids stress. J. Hazard. Mater..

[8] Bielach A., Hrtyan M., Tognetti V.B. (2017). Plants under stress: Involvement of auxin and cytokinin. Int. J. Mol. Sci..

[9] Casanova-Saez R., Voss U. (2019). Auxin metabolism controls developmental decisions in land plants. Trends Plant Sci..

[10] Hofmann N.R. (2011). YUC and TAA1/TAR proteins function in the same pathway for auxin biosynthesis. Plant Cell.

[11] Sugawara S., Hishiyama S., Jikumaru Y., Hanada A., Nishimura T., Koshiba T., Zhao Y., Kamiya Y., Kasahara H. (2009). Biochemical analyses of indole-3-acetaldoxime-dependent auxin biosynthesis in *Arabidopsis*. Proc. Natl. Acad. Sci. USA.

[12] Morffy N., Strader L.C. (2020). Old Town Roads: Routes of auxin biosynthesis across kingdoms. Curr. Opin. Plant Biol..

[13] Novak O., Henykova E., Sairanen I., Kowalczyk M., Pospisil T., Ljung K. (2012). Tissue-specific profiling of the *Arabidopsis thaliana* auxin metabolome. Plant J..

[14] Cao X., Yang H., Shang C., Ma S., Liu L., Cheng J. (2019). The roles of auxin biosynthesis YUCCA gene family in plants. Int. J. Mol. Sci..

[15] Zhao Y. (2018). Essential roles of local auxin biosynthesis in plant development and in adaptation to environmental changes. Annu. Rev. Plant Biol..

[16] Mano Y., Nemoto K. (2012). The pathway of auxin biosynthesis in plants. J. Exp. Bot..

[17] Harrison C.J. (2017). Auxin transport in the evolution of branching forms. New Phytol..

[18] Leyser O. (2018). Auxin signaling. Plant Physiol..

[19] Tan S., Luschnig C., Friml J. (2021). Pho-view of auxin: Reversible protein phosphorylation in auxin biosynthesis, transport and signaling. Mol. Plant.

[20] Lavy M., Estelle M. (2016). Mechanisms of auxin signaling. Development.

[21] Korasick D.A., Enders T.A., Strader L.C. (2013). Auxin biosynthesis and storage forms. J. Exp. Bot..

[22] Gonzalez A., Hall M.N. (2017). Nutrient sensing and TOR signaling in yeast and mammals. EMBO J..

[23] Gonzalez A., Hall M.N., Lin S.C., Hardie D.G. (2020). AMPK and TOR: The Yin and Yang of cellular nutrient sensing and growth control. Cell Metab..

[24] Hua H., Kong Q., Zhang H., Wang J., Luo T., Jiang Y. (2019). Targeting mTOR for cancer therapy. J. Hematol. Oncol..

[25] Weisman R. (2016). Target of rapamycin (TOR) regulates growth in response to nutritional signals. Microbiol. Spectr..

[26] Deng K., Wang W., Feng L., Yin H., Xiong F., Ren M. (2020). Target of rapamycin regulates potassium uptake in *Arabidopsis* and potato. Plant Physiol. Biochem..

[27] Song Y., Alyafei M.S., Masmoudi K., Jaleel A., Ren M. (2021). Contributions of TOR signaling on photosynthesis. Int. J. Mol. Sci..

[28] Dong P., Xiong F., Que Y., Wang K., Yu L., Li Z., Ren M. (2015). Expression profiling and functional analysis reveals that TOR is a key player in regulating photosynthesis and phytohormone signaling pathways in *Arabidopsi*. Front. Plant Sci..

[29] Fu L., Wang P., Xiong Y. (2020). Target of rapamycin signaling in plant stress responses. Plant Physiol..

[30] Wu Y., Shi L., Li L., Fu L., Liu Y., Xiong Y., Sheen J. (2019). Integration of nutrient, energy, light, and hormone signalling via TOR in plants. J. Exp. Bot..

[31] Bakshi A., Moin M., Madhav M.S., Kirti P.B. (2019). Target of rapamycin, a master regulator of multiple signalling pathways and a potential candidate gene for crop improvement. Plant Biol..

[32] Zhao Y., Wang X.Q. (2020). The hot issue: TOR signalling network in plants. Funct. Plant Biol..

[33] Ren M., Venglat P., Qiu S., Feng L., Cao Y., Wang E., Xiang D., Wang J., Alexander D., Chalivendra S. (2012). Target of rapamycin signaling regulates metabolism, growth, and life span in *Arabidopsis*. Plant Cell.

[34] Menand B., Desnos T., Nussaume L., Berger F., Bouchez D., Meyer C., Robaglia C. (2002). Expression and disruption of the *Arabidopsis* TOR (target of rapamycin) gene. Proc. Natl. Acad. Sci. USA.

[35] Deprost D., Yao L., Sormani R., Moreau M., Leterreux G., Nicolai M., Bedu M., Robaglia C., Meyer C. (2007). The *Arabidopsis* TOR kinase links plant growth, yield, stress resistance and mRNA translation. EMBO Rep..

[36] Caldana C., Li Y., Leisse A., Zhang Y., Bartholomaeus L., Fernie A.R., Willmitzer L., Giavalisco P. (2013). Systemic analysis of inducible target of rapamycin mutants reveal a general metabolic switch controlling growth in *Arabidopsis thalian*. Plant J..

[37] Song Y., Li L., Yang Z., Zhao G., Zhang X., Wang L., Zheng L., Zhuo F., Yin H., Ge X. (2018). Target of rapamycin (TOR) regulates the expression of incRNAs in response to abiotic stresses in cotton. Front. Genet..

[38] Deng K., Dong P., Wang W., Feng L., Xiong F., Wang K., Zhang S., Feng S., Wang B., Zhang J. (2017). The TOR pathway is involved in adventitious root formation in *Arabidopsis* and potato. Front. Plant Sci..

[39] Xiong F., Zhang R., Meng Z., Deng K., Que Y., Zhuo F., Feng L., Guo S., Datla R., Ren M. (2017). Brassinosteriod Insensitive 2 (BIN2) acts as a downstream effector of the Target of Rapamycin (TOR) signaling pathway to regulate photoautotrophic growth in Arabidopsis. New Phytol..

[40] Mahfouz M.M., Kim S., Delauney A.J., Verma D.P. (2006). *Arabidopsis* TARGET OF RAPAMYCIN interacts with RAPTOR, which regulates the activity of S6 kinase in response to osmotic stress signals. Plant Cell.

[41] Brumos J., Robles L.M., Yun J., Vu T.C., Jackson S., Alonso J.M., Stepanova A.N. (2018). Local auxin biosynthesis is a key regulator of plant development. Dev. Cell.

[42] Olatunji D., Geelen D., Verstraeten I. (2017). Control of endogenous auxin levels in plant root development. Int. J. Mol. Sci..

[43] Quilichini T.D., Gao P., Pandey P.K., Xiang D., Ren M., Datla R. (2019). A role for TOR signaling at every stage of plant life. J. Exp. Bot..

[44] De Vleesschauwer D., Filipe O., Hoffman G., Seifi H.S., Haeck A., Canlas P., Van Bockhaven J., De Waele E., Demeestere K., Ronald P. (2018). Target of rapamycin signaling orchestrates growth-defense trade-offs in plants. New Phytol..

[45] Schepetilnikov M., Ryabova L.A. (2018). Recent discoveries on the role of TOR (Target of Rapamycin) signaling in translation in plants. Plant Physiol..

[46] Zhu T., Li L., Feng L., Mo H., Ren M. (2020). Target of rapamycin regulates genome methylation reprogramming to control plant growth in *Arabidopsis*. Front. Genet..

[47] Bogre L., Henriques R., Magyar Z. (2013). TOR tour to auxin. EMBO J..

[48] Retzer K., Weckwerth W. (2021). The TOR-auxin connection upstream of root hair growth. Plants.

[49] Deng K., Yu L., Zheng X., Zhang K., Wang W., Dong P., Zhang J., Ren M. (2016). Target of rapamycin is a key player for auxin signaling transduction in *Arabidopsis*. Front. Plant Sci..

[50] Armenta-Medina A., Gillmor C.S., Gao P., Mora-Macias J., Kochian L.V., Xiang D., Datla R. (2021). Developmental and genomic architecture of plant embryogenesis: From model plant to crops. Plant Commun..

[51] Wang K., Chen H., Miao Y., Bayer M. (2020). Square one: Zygote polarity and early embryogenesis in flowering plants. Curr. Opin. Plant Biol..

[52] Dresselhaus T., Jurgens G. (2021). Comparative embryogenesis in angiosperms: Activation and patterning of embryonic cell lineages. Annu. Rev. Plant Biol..

[53] Ponce M.R., Micol J.L. (2020). A cornucopia of mutants for understanding plant embryo development. New Phytol..

[54] Verma S., Attuluri S., Robert H.S. (2021). An essential function for auxin in embryo development. CSH Perspect. Biol..

[55] Robert H.S., Park C., Gutierrez C.L., Wojcikowska B., Pencik A., Novak O., Chen J., Grunewald W., Dresselhaus T., Friml J. (2018). Maternal auxin supply contributes to early embryo patterning in *Arabidopsis*. Nat. Plants.

[56] Wang B., Liu G., Zhang J., Li Y., Yang H., Ren D. (2018). The RAF-like mitogen-activated protein kinase kinase kinases RAF22 and RAF28 are required for the regulation of embryogenesis in *Arabidopsis*. Plant J..

[57] Schlereth A., Moller B., Liu W., Kientz M., Flipse J., Rademacher E.H., Schmid M., Jurgens G., Weijers D. (2010). MONOPTEROS controls embryonic root initiation by regulating a mobile transcription factor. Nature.

[58] Ohashi-Ito K., Iwamoto K., Nagashima Y., Kojima M., Sakakibara H., Fukuda H. (2019). A positive feedback loop comprising LHW-TMO5 and local auxin biosynthesis regulates initial vascular development in *Arabidopsis* roots. Plant Cell Physiol..

[59] Gundu S., Tabassum N., Blilou I. (2020). Moving with purpose and direction: Transcription factor movement and cell fate determination revisited. Curr. Opin. Plant Biol..

[60] Wolters H., Jurgens G. (2009). Survival of the flexible: Hormonal growth control and adaptation in plant development. Nat. Rev. Genet..

[61] Zhang Y., Jiao Y., Jiao H., Zhao H., Zhu Y.X. (2017). Two-step functional innovation of the stem-cell factors WUS/WOX5 during plant evolution. Mol. Biol. Evol..

[62] Zhou Y., Liu X., Engstrom E.M., Nimchuk Z.L., Pruneda-Paz J.L., Tarr P.T., Yan A., Kay S.A., Meyerowitz E.M. (2015). Control of plant stem cell function by conserved interacting transcriptional regulators. Nature.

[63] Kong X., Lu S., Tian H., Ding Z. (2015). WOX5 is shining in the root stem cell niche. Trends Plant Sci..

[64] Scheres B., Krizek B.A. (2018). Coordination of growth in root and shoot apices by AIL/PLT transcription factors. Curr. Opin. Plant Biol..

[65] Smit M.E., Weijers D. (2015). The role of auxin signaling in early embryo pattern formation. Curr. Opin. Plant Biol..

[66] Moller B., Weijers D. (2009). Auxin control of embryo patterning. CSH Perspect Biol..

[67] Deprost D., Truong H.N., Robaglia C., Meyer C. (2005). An *Arabidopsis* homolog of RAPTOR/KOG1 is essential for early embryo development. Biochem. Biophys. Res. Commun..

[68] Salem M.A., Li Y., Wiszniewski A., Giavalisco P. (2017). Regulatory-associated protein of TOR (RAPTOR) alters the hormonal and metabolic composition of Arabidopsis seeds, controlling seed morphology, viability and germination potential. Plant J..

[69] Obomighie I., Lapenas K., Murphy B.E., Bowles A.M.C., Bechtold U., Prischi F. (2021). The role of ribosomal protein S6 kinases in plant homeostasis. Front. Mol. Biosci..

[70] Pardal R., Heidstra R. (2021). Root stem cell niche networks: It’s complexed! A review on gene networks regulating the *Arabidopsis*. J. Exp. Bot..

[71] Naramoto S. (2017). Polar transport in plants mediated by membrane transporters: Focus on mechanisms of polar auxin transport. Curr. Opin. Plant Biol..

[72] Barbosa I.C.R., Hammes U.Z., Schwechheimer C. (2018). Activation and polarity control of PIN-FORMED auxin transporters by phosphorylation. Trends Plant Sci..

[73] Lee H.J., Kim H.S., Park J.M., Cho H.S., Jeon J.H. (2020). PIN-mediated polar auxin transport facilitates root-obstacle avoidance. New Phytol..

[74] Sauer M., Kleine-Vehn J. (2019). PIN-FORMED and PIN-LIKES auxin transport facilitators. Development.

[75] Swarup R., Bhosale R. (2019). Developmental roles of AUX1/LAX auxin influx carriers in plants. Front. Plant Sci..

[76] Zwiewka M., Bilanovicova V., Seifu Y.W., Nodzynski T. (2019). The nuts and bolts of PIN auxin efflux carriers. Front. Plant Sci..

[77] Shimotohno A., Heidstra R., Blilou I., Scheres B. (2018). Root stem cell niche organizer specification by molecular convergence of PLETHORA and SCARECROW transcription factor modules. Genes Dev..

[78] Liebsch D., Palatnik J.F. (2020). MicroRNA miR396, GRF transcription factors and GIF co-regulators: A conserved plant growth regulatory module with potential for breeding and biotechnology. Curr. Opin. Plant Biol..

[79] Guillotin B., Birnbaum K.D. (2020). Just passing through: The auxin gradient of the root meristem. Curr. Top Dev. Biol..

[80] Santuari L., Sanchez-Perez G.F., Luijten M., Rutjens B., Terpstra I., Berke L., Gorte M., Prasad K., Bao D., Timmermans-Hereijgers J.L. (2016). The PLETHORA gene regulatory network guides growth and cell differentiation in Arabidopsis roots. Plant Cell.

[81] Xu T., Nagawa S., Yang Z. (2011). Uniform auxin triggers the Rho GTPase-dependent formation of interdigitation patterns in pavement cells. Small GTPases.

[82] Schepetilnikov M., Makarian J., Srour O., Geldreich A., Yang Z., Chicher J., Hammann P., Ryabova L.A. (2017). GTPase ROP2 binds and promotes activation of target of rapamycin, TOR, in response to auxin. EMBO J..

[83] Schepetilnikov M., Dimitrova M., Mancera-Martinez E., Geldreich A., Keller M., Ryabova L.A. (2013). TOR and S6K1 promote translation reinitiation of uORF-containing mRNAs via phosphorylation of eIF3h. EMBO J..

[84] Ahmad Z., Magyar Z., Bogre L., Papdi C. (2019). Cell cycle control by the target of rapamycin signalling pathway in plants. J. Exp. Bot..

[85] Forzani C., Aichinger E., Sornay E., Willemsen V., Laux T., Dewitte W., Murray J.A. (2014). WOX5 suppresses *CYCLIN D* activity to establish quiescence at the center of the root stem cell niche. Curr. Biol..

[86] Yuan X., Xu P., Yu Y., Xiong Y. (2020). Glucose-TOR signaling regulates PIN2 stability to orchestrate auxin gradient and cell expansion in *Arabidopsis* root. Proc. Natl. Acad. Sci. USA..

[87] Motte H., Beeckman T. (2019). The evolution of root branching: Increasing the level of plasticity. J. Exp. Bot..

[88] Chiatante D., Rost T., Bryant J., Scippa G.S. (2018). Regulatory networks controlling the development of the root system and the formation of lateral roots: A comparative analysis of the roles of pericycle and vascular cambium. Ann. Bot..

[89] Torres-Martinez H.H., Rodriguez-Alonso G., Shishkova S., Dubrovsky J.G. (2019). Lateral root primordium morphogenesis in *angiosperms*. Front. Plant Sci..

[90] Torres-Martinez H.H., Hernandez-Herrera P., Corkidi G., Dubrovsky J.G. (2020). From one cell to many: Morphogenetic field of lateral root founder cells in *Arabidopsis thaliana* is built by gradual recruitment. Proc. Natl. Acad. Sci. USA..

[91] Zhang Y., Mitsuda N., Yoshizumi T., Horii Y., Oshima Y., Ohme-Takagi M., Matsui M., Kakimoto T. (2021). Two types of bHLH transcription factor determine the competence of the pericycle for lateral root initiation. Nat. Plants.

[92] Du Y., Scheres B. (2018). Lateral root formation and the multiple roles of auxin. J. Exp. Bot..

[93] Liu W., Yu J., Ge Y., Qin P., Xu L. (2018). Pivotal role of *LBD16* in root and root-like organ initiation. Cell Mol. Life Sci..

[94] Soyano T., Shimoda Y., Kawaguchi M., Hayashi M. (2019). A shared gene drives lateral root development and root nodule symbiosis pathways in *Lotus*. Science.

[95] Schiessl K., Lilley J.L.S., Lee T., Tamvakis I., Kohlen W., Bailey P.C., Thomas A., Luptak J., Ramakrishnan K., Carpenter M.D. (2019). *NODULE INCEPTION* recruits the lateral root developmental program for symbiotic nodule organogenesis in *medicago truncatula*. Curr. Biol..

[96] Singh S., Yadav S., Singh A., Mahima M., Singh A., Gautam V., Sarkar A.K. (2020). Auxin signaling modulates *LATERAL ROOT PRIMORDIUM1* (*LRP1*) expression during lateral root development in *Arabidopsis*. Plant J..

[97] Du Y., Scheres B. (2017). PLETHORA transcription factors orchestrate de novo organ patterning during *Arabidopsis* lateral root outgrowth. Proc. Natl. Acad. Sci. USA..

[98] Teixeira J.S., Ten Tusscher K.H. (2019). The systems biology of lateral root formation: Connecting the dots. Mol. Plant.

[99] Berckmans B., Vassileva V., Schmid S.P., Maes S., Parizot B., Naramoto S., Magyar Z., Alvim Kamei C.L., Koncz C., Bogre L. (2011). Auxin-dependent cell cycle reactivation through transcriptional regulation of *Arabidopsis E2Fa* by lateral organ boundary proteins. Plant Cell.

[100] Magyar Z., Horvath B., Khan S., Mohammed B., Henriques R., De Veylder L., Bako L., Scheres B., Bogre L. (2012). *Arabidopsis* E2FA stimulates proliferation and endocycle separately through RBR-bound and RBR-free complexes. EMBO J..

[101] Nanjareddy K., Blanco L., Arthikala M.K., Alvarado-Affantranger X., Quinto C., Sanchez F., Lara M. (2016). A legume TOR protein kinase regulates rhizobium symbiosis and is essential for infection and nodule development. Plant Physiol..

[102] Mendez-Gomez M., Castro-Mercado E., Pena-Uribe C.A., Reyes-de la Cruz H., Lopez-Bucio J., Garcia-Pineda E. (2020). TARGET OF RAPAMYCIN signaling plays a role in *Arabidopsis* growth promotion by *Azospirillum brasilense* Sp245. Plant Sci..

[103] Arthikala M.K., Nanjareddy K., Blanco L., Alvarado-Affantranger X., Lara M. (2021). Target of rapamycin, *PvTOR*, is a key regulator of arbuscule development during mycorrhizal symbiosis in *Phaseolus*. Sci. Rep..

[104] Lardon R., Geelen D. (2020). Natural variation in plant pluripotency and regeneration. Plants.

[105] Xu L. (2018). *De novo* root regeneration from leaf explants: Wounding, auxin, and cell fate transition. Curr. Opin. Plant Biol..

[106] Yu J., Liu W., Liu J., Qin P., Xu L. (2017). Auxin control of root organogenesis from callus in tissue culture. Front. Plant Sci..

[107] Liu J., Sheng L., Xu Y., Li J., Yang Z., Huang H., Xu L. (2014). *WOX11* and *12* are involved in the first-step cell fate transition during *de novo* root organogenesis in *Arabidopsis*. Plant Cell.

[108] Hu X., Xu L. (2016). Transcription factors WOX11/12 directly activate *WOX5/7* to promote root primordia initiation and organogenesis. Plant Physiol..

[109] Liu J., Hu X., Qin P., Prasad K., Hu Y., Xu L. (2018). The *WOX11-LBD16* pathway promotes pluripotency acquisition in callus cells during de novo shoot regeneration in tissue culture. Plant Cell Physiol..

[110] Street I.H., Mathews D.E., Yamburkenko M.V., Sorooshzadeh A., John R.T., Swarup R., Bennett M.J., Kieber J.J., Schaller G.E. (2016). Cytokinin acts through the auxin influx carrier AUX1 to regulate cell elongation in the root. Development.

[111] Shu W., Zhou H., Jiang C., Zhao S., Wang L., Li Q., Yang Z., Groover A., Lu M.Z. (2019). The auxin receptor TIR1 homolog (PagFBL 1) regulates adventitious rooting through interactions with Aux/IAA28 in *Populus*. Plant Biotechnol. J..

[112] Zhang X., Wang G., Zhang S., Chen S., Wang Y., Wen P., Ma X., Shi Y., Qi R., Yang Y. (2020). Genomes of the banyan tree and pollinator wasp provide insights into Fig-Wasp coevolution. Cell.

